# Clinical and laboratory profile of patients with TB/HIV coinfection: A case series of 50 patients

**DOI:** 10.4103/0970-2113.80316

**Published:** 2011

**Authors:** Anand K. Patel, Sandip J. Thakrar, Feroz D. Ghanchi

**Affiliations:** *Department of Pulmonary Medicine, S.B.K.S. Medical Institute and Research Centre, Sumandeep Vidhyapeeth University, Piparia, Vadodara, Gujarat, India*; 1 *Tuberculosis and Chest Diseases, M. K. Thakrar Hospital, Porbandar, Gujarat, India*; 2 *Pulmonary Medicine, Shree M. P. Shah Medical College, Guru Gobindsing Hospital, Jamnagar, Gujarat, India*

**Keywords:** HIV, signs and symptoms, tuberculosis

## Abstract

**Background:**

Tuberculosis (TB) is said to be one of the commonest opportunistic infection in patients with HIV/AIDS.

**Objective:**

To study the clinical and laboratory profile of patients with HIV/TB coinfection.

**Materials and Methods:**

Fifty adult TB patients having confirmed HIV seropositivity were included in randomized manner. A detailed history and thorough physical examination was done. Laboratory and radiological investigations were carried out as appropriately warranted.

**Results:**

Most of the patients were farm workers (30%) followed by manual laborers (22%) and transport drivers (16%). Heterosexual route was found in 86% of patients. Cough was present in 94% while fever and weight loss in 86% and 78% of patients, respectively. Out of 50 patients, 40% had only pulmonary TB (PTB), 46% had pulmonary and extra-pulmonary TB (EPTB), 10% had only EPTB and 4% had multisystemic EPTB. Mediastinal lymphadenopathy was present in 34% while pleural effusion and extra-thoracic lymph nodes was present in 20% and 18% of patients, respectively. Positive smear for acid-fast bacilli (AFB) was found in 25.58% while positive Mantoux test was found in 32.14% of patients.

**Conclusion:**

HIV/TB coinfection is more common in sexually active age group and commonest mode of HIV infection is heterosexual transfer. Sputum smear AFB and Mantoux test positivity is low in TB patients having HIV. Disseminated TB is common in HIV. Mediastinal lymphadenopathy is common site among extra-pulmonary tuberculosis.

## INTRODUCTION

Tuberculosis remains the most common opportunistic infection (OI) and is the commonest cause of death in HIV infected patients.[1] Clinical presentation of TB in early HIV infection resembles that observed in immuno-competent persons. In late HIV infection, the clinical presentation of TB can be atypical. Diagnosis of TB in HIV infected patients may be delayed because of atypical clinical presentation and involvement of inaccessible sites and low sputum smear positivity.[2] HIV-infected individuals have an extraordinarily high risk of developing clinical TB gives cause for serious concern as the implications are most serious.[2]

In 2008, there were estimated 9.4 million new cases of TB globally; of which 1.4 million (15%) were HIV positive. An estimated 1.8 million people died of TB globally, of which about 0.5 million were patients with TB/HIV coinfection.[3]

In 2008, 1.98 million TB cases were estimated to have occurred in India with 276 000 (24/100 000 population) TB mortality. India, the third highest HIV burdened country, had an estimated 2.31 million (0.36% of adult population in the country) people living with HIV/AIDS (PLHAs) in 2007. It has been estimated that in 2007, about 4.85% of the incident of TB cases in India were HIV-positive.[3]

In persons dually infected with HIV and tuberculosis, the lifetime risk of developing tuberculosis is 50%–70% as compared to a 10% risk in HIV negative individuals.[4] Due to this relationship there has been a dramatic increase in the incidence of tuberculosis in countries with high prevalence of HIV and tuberculosis.[5]

Thus, because of the very frequent association of tuberculosis and HIV, it has become necessary to look for tuberculosis in HIV infected individuals and *vice versa*.

In this paper, we describe the clinical and laboratory findings from a case series of patients with TB/HIV coinfection, in order to obtain a better picture of the clinical profile of these patients.

## MATERIALS AND METHODS

In the present study 50 adult TB patients having confirmed HIV sero-positivity either attending OPD or hospitalized in Guru Gobindsing Hospital and Shree M. P. Shah Medical College, Jamnagar, Gujarat; were selected in randomized manner. A detailed clinical history and complete general physical and systemic examination findings were recorded. All patients were subjected to chest radiograph and sputum smear AFB examination of three samples. Mantoux test using 5 TU of purified protein derivative (PPD) was done and transverse diameter of induration was noted in mm in 28 patients.

### Diagnosis of HIV status

All the study patients were initially screened by HIV Tridot (J. Mitra. and Co., India) and if found positive on screening were confirmed by Immunocomb Bispot (Orgenic Ltd., Israel) and GENEDIA HIV ELISA (Green cross life science, Korea). All kits detect antibodies.

### Diagnosis of TB

Apart from clinical manifestations and history of contact with TB patient, the diagnosis of TB was based on (i) sputum smear AFB examination, (ii) chest radiograph, (iii) Mantoux test, (iv) fluid analysis and studies, and (v) response to antituberculosis treatment (ATT). Tissue biopsy/Fine needle aspiration cytology (FNAC) was carried out by obtaining relevant samples in cases of extra-pulmonary TB (EPTB). Antituberculosis treatment on clinical ground was given in suspected TB cases.

Other suggestive investigations were also carried out as and when required to establish the diagnosis of TB.

## RESULTS

Age distribution of the patients is shown in Table 1. There were 41 (82%) males and nine (18%) females. Thirty percent of patients were farmers while manual laborers and transport drivers accounted for 22% and 16%, respectively. Thirty-eight patients (76%) were married (among which four had second marriage), eight (16%) were unmarried, two (4%) were widow, one (2%) was widower, and one (2%) was divorced. Probable route of HIV transmission was as shown in Table 2. Among five patients with blood borne mode of transmission three (6%) had blood transfusion related, one (2%) had history of sharing infected shaving instrument, and one (2%) had history of intravenous medication. Out of 43 patients with history of heterosexual contacts, 21 (48.83%) had history of multiple frequent visits to commercial sex workers (CSW); 10 (23.27%) had history of extramarital affairs while six (13.95%) had both the above histories. Six (13.95%) female patients had acquired the infection probably by heterosexual contact with infected spouse. In overall 37 (86.05%) patients with high-risk heterosexual activities had multiple sex partners. History of unprotected sex was not authentically revealed by majority of patients with heterosexual contacts.

**Table 1:** Age distribution of the patients

Age group (range in years)	No. of patients (*n* = 50)	(%)
11–20	1	02
21–30	16	32
31–40	22	44
41–50	10	20
>50	1	2
Total	50	100

**Table 2:** Probable route of HIV transmission

Probable route of HIV transmission	No. of patients (*n* =50)	(%)
Heterosexual	43	86
Blood-borne	5	10
Homosexual	1	2
Undetermined	1	2
Total	50	100

Cough was the most common symptom present in 47 (94%) patients followed by fever, weight loss, and loss of appetite present in 43 (86%), 39 (78%), and 31 (62%) patients, respectively, while dyspnoea, chest pain, and hemoptysis were seen in 28 (56%), 10 (20%), and seven (14%) patients, respectively. The mean duration of the most common presenting symptom (cough) was 12 weeks while fever and weight loss had mean duration of about 14 and 12 weeks, respectively, at the time of presentation. Mean duration of anorexia was 15 weeks and for dyspnoea it was about 8 weeks. The average (mean) duration of symptoms at the time of presentation was 12.2 weeks, which is in overall suggestive of late presentation and contributing to the delay in the diagnosis of TB. The duration of illness in the present study ranged from 2 weeks to 2 years.

In the present study, only pulmonary TB (PTB) was seen in 20 (40%) patients, while only EPTB was seen in five (10%) patients (three – pleural, one – lymph node, one – CNS). Overall pulmonary involvement of TB was seen in 43 (86%) cases; extra-pulmonary involvement was seen in 30 (60%) cases while disseminated TB accounted for 25 (50%) cases. Out of 43 TB patients having pulmonary involvement sputum smear AFB was positive only in 11 (25.58%). Out of 11 sputum positive patients, six cases (54.55%) had 1+ positivity; two (18.18%) had 2+ positivity; two (18.18%) had 3+ positivity while scanty bacilli were seen in one (9.09%). Among 32 sputum negative cases, 20 patients had disseminated disease suggestive of late phase of immune-suppression, accounting for higher sputum AFB negativity. Mantoux test was done in 28 patients and its results are shown in Table 3.

**Table 3:** Distribution of Mantoux reaction

Induration range (in mm)	No. of patients (*n* = 28)	(%)
0–5	06	21.43
6–10	13	46.43
11–20	09	32.14
Total	28	100

Pulmonary tuberculosis was the commonest form of TB observed in 43 (86%) patients. Among EPTB mediastinal lymphadenopathy was seen in 17 (34%) patients followed by pleural effusion seen in 10 (20%) patients. Other commoner forms of EPTB were extra-thoracic adenopathy and abdominal (peritoneal and GIT) TB seen in nine (18%), and six (12%) patients, respectively. Other forms included pericardial effusion (6%), CNS TB (6%). Disseminated TB was seen in 25 (50%) patients.

## DISCUSSION

After the detection and recognition of HIV in 1983, the declining curve of TB infection started to show a sudden rise during the 1990s. Coinfection with TB and HIV has already been reported as one of the most significant global public health concerns.[6] Tuberculosis is the commonest opportunistic disease in HIV positive persons in India.[1] HIV/AIDS pandemic has caused a resurgence of TB, resulting in increased morbidity and mortality worldwide.[7] From the epidemiological point of view, our TB/HIV patients differed in some respects from those present in other parts of the world.

Most of our study group patients (76%) belonged to the age group of 21–40 years, which is the sexually active age and is also the most productive in one’s life. Of all the detected patients, 82% were males and the rest were females. The striking male predominance noted in the present study has also been reported by other authors.[8–10] The occupational profile of our patients revealed that a majority of them were farmers and laborers followed by transport drivers. Mohanty *et al*.[11] reported 36.8% patients working as manual laborers while Rajsekaran *et al*.[12] found majority (55.6) of patients working as farmer. Other authors[9] have found sero-positivity rate was highest among those who were unemployed (40%) followed by the business professionals (35%). The percentage of the professions is thus seen to vary in different studies, largely due to the differences in the occupational patterns and the source from where the patients were selected.

Sexual route (heterosexual) was found to be the major risk factor (86%) while only one patient was an intravenous drug abuser and one patient was homosexual in our study. Three patients (6%) had blood-transfusion-related transmission. Heterosexual promiscuity and casual sex was found to be a major risk factor in the studies by some Indian observers[10,11] while other[9] observed that the majority of their cases were intravenous drug abusers (68.9%).

The average duration of symptoms was 12.2 weeks, indicating that there was a delay in diagnosing tuberculosis and starting treatment. Whether the delay was at the patient or provider level needs further investigation. The duration of illness in our patients ranged from 2 weeks to 2 years. Swaminathan *et al*.[10] found that the duration of illness in their cases before seeking treatment was 12 weeks.

Tuberculin test positivity (>10 mm) to 5 TU PPD was observed in 32.14% patients in our study. Other authors have reported a wide variation in tuberculin test positivity.[9,11] Positive response to tuberculin is generally retained early in the course of HIV infection.

The most common symptom was cough in 47 (94%) patients, while fever was present in 43 (86%) and weight loss in 39 (78%) patients. In the series reported by Mohanty *et al*.[11] fever was the most common complaint, while Deivanayagam *et al*.[8] reported cough with expectoration in majority of their patients.

Eleven (25.58%) of our patients had sputum smear for AFB positive. This is very different from the situation in HIV uninfected tuberculosis patients and indicates that smear microscopy is not a sensitive diagnostic tool in the presence of HIV infection. Mohanty *et al*.[11] has reported 31.59% while Deivanayagam *et al*.[8] has reported 15% patients as smear positive. It has been shown that sputum smear is often positive in the early stage of HIV infection.[4]

Extra-pulmonary tuberculosis is more common in HIV/TB patients, especially with advanced immunesuppression than in non-HIV/TB patients.[4] Extra-pulmonary tuberculosis was seen in 30 (60%) of our HIV/TB patients. Most common form of extra-pulmonary TB was mediastinal lymphadenopathy followed by pleural effusion and extra-thoracic adenopathy. Overall involvement of lymphatic system was seen in 21 (42%) patients. Other authors have also observed that lymphatic system is the most commonly involved, followed by pleural involvement in HIV/TB patients.[4,12]

Twenty-three patients (53.49%) out of 43 PTB patients had an associated extra-pulmonary focus, and two patients with extra-pulmonary TB had multisystem involvement which is pointing to the disseminated nature of the disease in HIV positive.

We conclude that HIV/TB coinfection is more common in sexually active age group and heterosexual transfer is the commonest mode of HIV infection. Sputum smear AFB and Mantoux test positivity is low in TB patients having HIV. Disseminated TB is common in HIV and mediastinal lymphadenopathy is common site among extra-pulmonary tuberculosis.
